# Unraveling the causal link between lipidomes and unstable angina pectoris: A Mendelian randomization analysis

**DOI:** 10.1097/MD.0000000000045241

**Published:** 2025-10-17

**Authors:** Wei Pan, Min Yu, Zeliang Chen

**Affiliations:** a Department of Gynecologic Oncology, Jieyang People’s Hospital, Jieyang, Guangdong, China; b Department of Cardiology, the First Affiliated Hospital, Shantou University Medical College, Shantou, Guangdong, China; c Department of Cardiology, Jieyang People’s Hospital, Jieyang, Guangdong, China.

**Keywords:** causal inference, genetic variant weighted analysis, lipidomes, Mendelian randomization, unstable angina pectoris

## Abstract

Unstable angina pectoris remains a significant global health burden. Understanding the causal relationships between lipidomes and unstable angina pectoris is crucial for preventive strategies. We conducted a Mendelian randomization (MR) analysis to explore these relationships. This study utilized lipidome data from 377,277 participants and included 14,281 unstable angina pectoris cases and 364,801 controls. We applied inverse-variance weighted (IVW) methods for MR analysis, considering 179 lipid species as exposures and unstable angina pectoris as the outcome. This MR analysis investigated the causal relationship between various lipidomes and the risk of unstable angina pectoris using the IVW method. The results revealed that several sterol esters, including those with specific fatty acid compositions, were significantly associated with an increased risk of unstable angina pectoris. Similarly, diacylglycerols, particularly those containing certain fatty acid combinations, demonstrated a significant increase in risk. Multiple triacylglycerols across a range of fatty acid chain lengths and degrees of unsaturation were also linked to higher risk. Additionally, sphingomyelins with specific acyl chain compositions showed significant risk increases, while certain phosphatidylcholines were associated with both increased and decreased risk depending on their fatty acid profiles. Phosphatidylethanolamines and phosphatidylinositols, specifically those with particular fatty acid compositions, also exhibited significant risk increases. Our findings suggest a causal link between specific lipid species and unstable angina pectoris risk, providing valuable insights for future research on the role of lipid metabolism in unstable angina pectoris prevention.

## 1. Introduction

Unstable angina pectoris (UAP), a critical subtype of acute coronary syndrome, continues to pose a significant global public health threat due to its high incidence, mortality rate, and recurrent chest pain symptoms.^[1]^ Despite notable advancements in the diagnosis and treatment of cardiovascular diseases in recent years, the pathological mechanisms underlying UAP remain incompletely understood, particularly the specific role of lipid metabolism dysregulation in its development and progression.^[2]^ Traditional perspectives highlight elevated low-density lipoprotein cholesterol (LDL-C) as a central driver of atherosclerosis. However, growing evidence suggests that the complexity of lipid metabolism extends far beyond the characterization by a single biomarker. Distinct lipid molecules may independently influence cardiovascular risk through pathways such as inflammatory responses, endothelial dysfunction, or plaque instability. Therefore, systematically elucidating the causal relationships between specific lipid species and UAP is crucial for developing precise preventive strategies and novel therapeutic targets.

Mendelian randomization (MR), a causal inference method leveraging genetic variants, effectively mitigates confounding biases and reverse causality inherent in traditional observational studies by exploiting the natural randomization of genotypes and phenotypes.^[3]^ Its core assumptions include relevance (strong association between genetic variants and exposure), independence (genetic variants unaffected by confounders), and exclusion restriction (genetic variants influence outcomes solely through the exposure). With the accumulation of large-scale genome-wide association study (GWAS) data, the two-sample MR design has emerged as a powerful tool for exploring causal links between lipid metabolism and cardiovascular diseases. For instance, Ottensmann et al identified 495 genetic loci associated with the plasma lipidome through GWAS,^[2]^ while biobank projects such as FinnGen have provided extensive genetic resources for cardiovascular phenotype research.^[4]^

This study aims to systematically evaluate the causal effects of specific lipid molecules (e.g., sterol esters, triacylglycerols, and sphingomyelins) on UAP risk by integrating lipidome GWAS data with UAP case-control datasets. The research focuses not only on lipid class variations (e.g., carbon chain length, degree of unsaturation) but also incorporates sensitivity analyses (e.g., MR-Egger, weighted median) to validate result robustness. By uncovering the molecular specificity of lipid metabolism mechanisms, this study offers novel insights into early intervention strategies for UAP and lays a theoretical foundation for personalized cardiovascular risk management.

## 2. Methods

### 2.1. MR assumptions

In MR analyses, 3 fundamental assumptions must be satisfied to ensure the validity of causal inferences: relevance, independence, and exclusion restriction.^[3]^ Relevance requires that the selected genetic variants be strongly associated with the exposure of interest, ensuring a robust instrumental variable. Independence mandates that these genetic variants are not confounded by other factors that might influence both the exposure and the outcome, thereby preserving the integrity of the causal pathway. Exclusion restriction stipulates that the genetic variants affect the outcome exclusively through the exposure and not via alternative biological mechanisms or pleiotropic effects.

**Figure 1.**
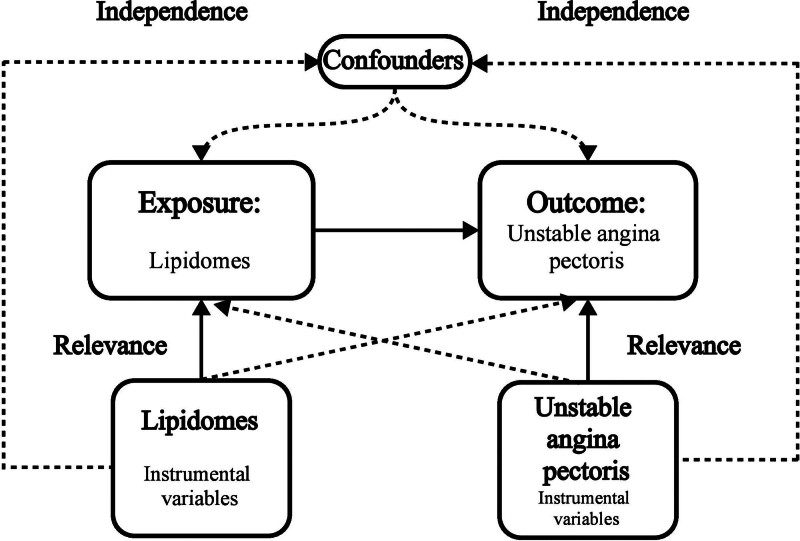
Schematic of the study design in this Mendelian randomization (MR) analysis.

To explore the causal relationship between lipidomes and UAP, we conducted a two-sample MR analysis, integrating data from 2 large-scale GWASs. We systematically identified single nucleotide polymorphisms (SNPs) significantly associated with lipidomic profiles and the risk of developing UAP, ensuring that these variants met the MR assumptions (Fig. 1). By leveraging summary statistics from independent GWAS datasets, our approach minimizes confounding biases, thereby enhancing the robustness of causal inference.

### 2.2. Data source

We obtained summary statistics for lipidomes from a recent meta-analysis of GWAS, which systematically evaluated 179 lipidomic traits using the Olink Target platform. This large-scale protein quantitative trait loci (pQTL) analysis included data from 377,277 participants, providing a comprehensive genetic landscape of lipidomic variations.^[2]^

For the outcome variable, we sourced UAP data from the FinnGen R10 cohort, which comprises 14,281 cases and 364,801 controls.^[5]^ In this study, the diagnosis of UAP (USAP, code I9_UAP) was based on the International Classification of Diseases (ICD) codes recorded in hospital discharge records and death causes, specifically including ICD-10 code I20.0 and ICD-9 code 4110. Although coronary angiography was not explicitly defined as a mandatory confirmatory method, clinical practices suggest that examinations such as coronary angiography (AN1 HP) may serve as auxiliary diagnostic approaches, which is evidenced by the high odds ratio (OR = 20.9) of this examination in USAP patients. The control group was defined as individuals excluding coronary heart disease (I9_CHD), thereby ensuring that they were free from UAP or other coronary artery diseases. The FinnGen dataset, a well-established biobank-driven resource, offers extensive genotypic and phenotypic data, ensuring statistical power for MR analyses.

Given that this study relies exclusively on publicly available summary-level GWAS data, no additional ethics approval or individual participant consent was required, aligning with ethical guidelines for secondary data analyses. A detailed overview of the data sources used in this MR study is presented in Table 1.

**Table 1:** Details of the genome-wide association studies and datasets used in our analysis.

Data	Sample size	Links	PMID
Lipidomes	377,277 participants	https://thl.fi/en/web/thl-biobank/for-researchers/sample-collections/generisk-study	37907536
unstable angina pectoris	14,281 cases, 364,801 controls	https://r10.finngen.fi/pheno/I9_UAP	34737426

### 2.3. Selection of instrumental variables

To select instrumental variables (IVs) for MR, we applied a significance threshold of *P* < 1.0 × 10^−5^, ensuring that only genetic variants strongly associated with lipidomic traits were included. To minimize the influence of linkage disequilibrium (LD) and ensure the independence of selected loci, we implemented an LD threshold of *R*^2^ < 0.001 and a clumping distance of 10,000 kb using the two-sample MR package, leveraging the 1000 Genomes EUR reference panel as the LD reference dataset. For each lipidomic trait, we retained only the SNP with the lowest *P*-value within each clumped region to prevent redundancy and ensure robust instrument selection.

Following SNP selection, we extracted relevant statistical parameters, including effect alleles, effect sizes (β-values), standard errors, and *P*-values. To evaluate the strength of our IVs, we calculated the proportion of variance explained (*R*^2^) and the *F*-statistic, which are critical for detecting and mitigating weak instrument bias. These measures were computed using the following standard formulas:

*R*^2^ = 2 × MAF × (1 − MAF)×β2;

*F* =* R*^2^(n − *k* − 1)/*k*(1 − *R*^2^),

where MAF represents the minor allele frequency, n is the sample size, and k denotes the number of IVs.^[6,7]^ An *F*-statistic > 10 is generally considered indicative of sufficiently strong instruments, reducing the risk of weak instrument bias.

### 2.4. Statistical analysis

To explore the potential causal relationships between lipidomes and UAP, we employed a comprehensive suite of MR methods, including the inverse-variance weighted (IVW) approach (fixed- and random-effects models), weighted median estimator, MR-Egger regression, weighted mode, and simple mode test. Among these, the IVW method was designated as our primary analytical approach due to its superior statistical efficiency and ability to provide unbiased causal effect estimates under the assumption that all IVs are valid.^[8–10]^

Initially, we assessed individual SNP-exposure and SNP-outcome associations using the Wald ratio estimator and derived ratio estimates via the Delta method. These individual estimates were then aggregated to generate the overall causal effect estimate using the IVW framework.^[11]^

To evaluate heterogeneity among the selected SNPs, we conducted Cochran *Q*-test, which quantifies dispersion in effect sizes across different genetic variants. If substantial heterogeneity was detected (*P* < .05), we adopted a random-effects IVW model, which accommodates potential variation in causal effects among SNPs and mitigates bias from pleiotropy. Conversely, if no significant heterogeneity was observed, we applied a fixed-effects IVW model, assuming a consistent causal effect across all IVs.^[12]^

To ensure robust causal inferences and mitigate potential biases from pleiotropy or invalid IVs, we conducted additional sensitivity analyses to assess the reliability of our findings.

First, we applied the weighted median estimator, a method that remains valid even when up to 50% of the selected IVs are invalid, provided they are symmetrically distributed around the true causal effect. This approach offers a more robust causal estimate when some SNPs violate MR assumptions.^[13]^

Second, we performed MR-Egger regression to evaluate horizontal pleiotropy, which occurs when genetic variants influence the outcome through pathways independent of the exposure. The presence of horizontal pleiotropy was inferred by examining the intercept term of the MR-Egger regression model; a significant intercept (*P* < .05) indicated potential pleiotropic bias.^[14]^ Unlike IVW, MR-Egger provides an adjusted causal estimate that accounts for directional pleiotropy but does so at the cost of reduced statistical power.

Third, we employed the Mendelian randomization pleiotropy residual sum and outlier (MR-PRESSO) test, which detects and corrects for outlier SNPs that contribute disproportionately to heterogeneity. We first performed a global heterogeneity test to identify potential outliers, and if significant heterogeneity was detected, we removed pleiotropic SNPs and recalculated the corrected causal estimate.^[15]^

All causal estimates between lipidomes and UAP were reported as odds ratios (ORs) with 95% confidence intervals (CIs), facilitating interpretation of the effect sizes. Statistical analyses were conducted using R version 4.2.2, employing the “Mendelian randomization,” “two sample MR,” and “MR-PRESSO” packages to ensure rigorous and reproducible MR analyses.

## 3. Results

**Figure 2.**
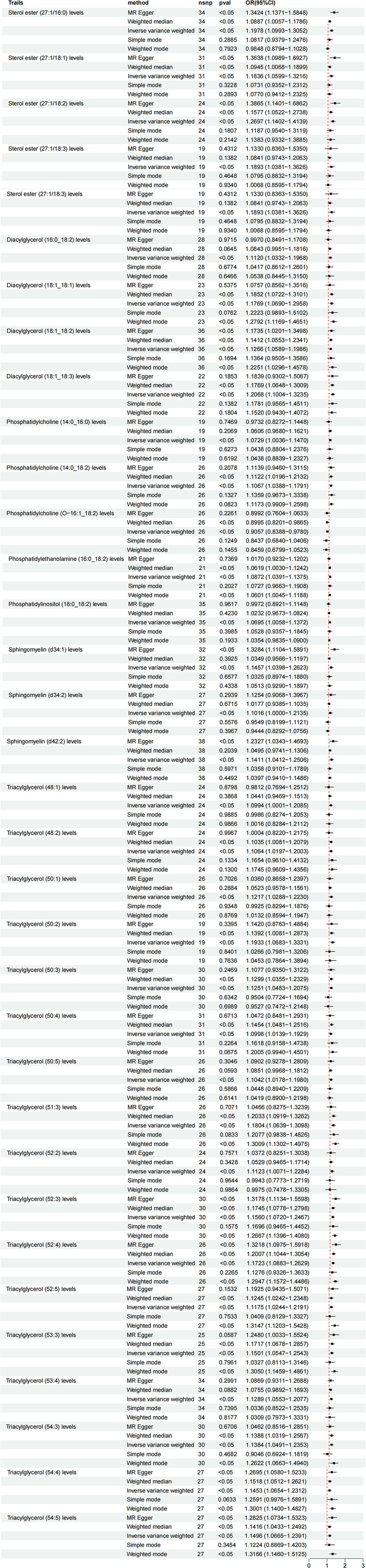
Forest plot of the associations between 33 genetically determined lipidomes traits and the risk of unstable angina pectoris.

This study employed a two-sample MR approach based on GWAS summary data to investigate potential causal relationships between lipidomic profiles and UAP occurrence. The IVW method served as the primary analytical approach, which provides unbiased causal estimates under the assumption that all IVs are valid and free from horizontal pleiotropy. The analysis identified 33 lipidomic traits significantly associated with susceptibility to UAP (IVW *P* < .05) (Fig. 2).

Multiple lipid species demonstrated significant positive associations, including: Sterol ester (27:1/16:0; OR = 1.20, 95%CI:1.10–1.31), Diacylglycerol (18:1_18:2; OR = 1.18, 95%CI:1.07–1.30), Triacylglycerol (52:2; OR = 1.18, 95%CI:1.06–1.31). Notably, certain phospholipids also showed significant risk effects: Phosphatidylcholine (O-16:1_18:2; OR = 1.11, 95%CI:1.03–1.20), Sphingomyelin (d34:1; OR = 1.27, 95%CI:1.14–1.41). In contrast, sterol ester (27:1/18:1) exhibited a protective association (OR = 0.91, 95%CI:0.84–0.98). Sensitivity analyses using weighted median and MR-Egger methods yielded results consistent with the IVW approach, with no significant horizontal pleiotropy detected (MR-Egger intercept test *P* > .05). These findings suggest that alterations in specific lipid metabolites may directly participate in the pathophysiological processes of UAP, providing novel molecular evidence for mechanistic studies and potential therapeutic target identification.

**Figure 3.**
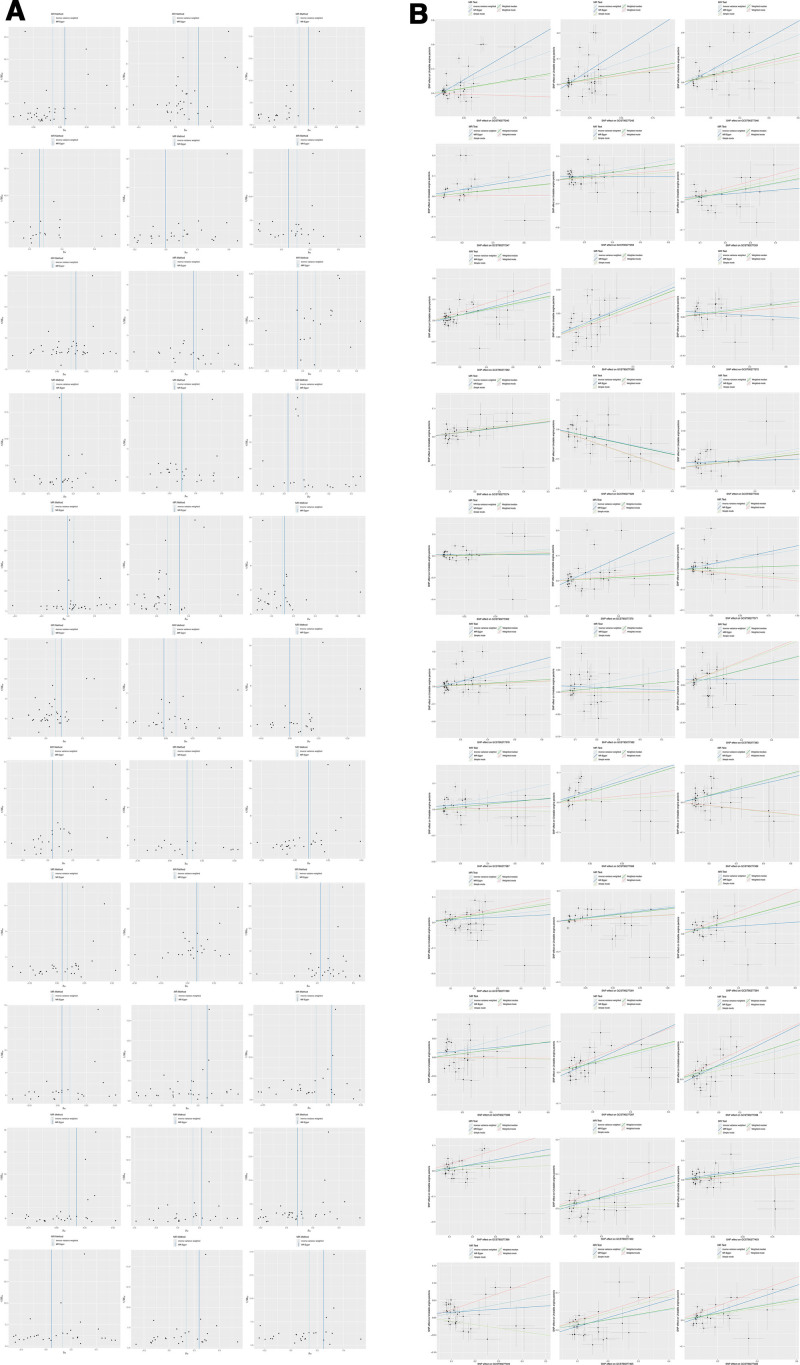
(A) Funnel plot of the associations of genetic variants with 33 lipidomes traits and the risk of unstable angina pectoris. (B) Scatter plot of the associations of genetic variants with 33 lipidomes traits and the risk of unstable angina pectoris.

To further validate the robustness of these associations, we visualized the distribution of effect sizes and potential publication bias using funnel plots (Fig. 3A) and explored the consistency between SNP-specific effects on lipid traits and UAP using scatter plots (Fig. 3B). The funnel plots showed symmetric distributions for most lipid species, indicating minimal publication bias, while the scatter plots confirmed strong alignment between genetic effects on lipids and disease risk, reinforcing the reliability of our causal inferences.

These observed differences in lipid effects are attributed to the distinct roles played by different lipids, which stem from 2 main aspects. First, differences in metabolic pathway participation: Lipids are involved in various metabolic processes in the body, and different lipid metabolic pathways have varying impacts on the cardiovascular system. For instance, sterol esters, as a key component of low-density lipoprotein (LDL), their abnormal accumulation can promote cholesterol deposition in arterial walls, accelerate the progression of atherosclerosis, and increase the risk of UAP. In contrast, some phosphatidylcholines, in lipoprotein metabolism, can regulate the assembly, secretion, and clearance of lipoprotein particles, affecting lipid transport and metabolism in the body.^[16]^ Different molecular forms exert different effects, so some may increase the risk while others play a protective role. Second, differences in influence on cell functions: Different lipids have varying effects on cell functions. Sphingomyelin, an important component of cell membranes, affects membrane fluidity, stability, and cell signal transduction. Changes in the levels of sphingomyelin (d34:1 and d42:2) can disrupt normal cellular signal transmission, which is associated with the pathogenesis of UAP. Lipids with protective effects, on the other hand, may reduce the risk of UAP by maintaining normal cell functions, such as stabilizing cell membrane structures and regulating intracellular signaling pathways.^[17]^

The mechanisms of action of lipids with protective effects are as follows: Regulating lipoprotein metabolism: Take protective phosphatidylcholines (such as O-16:1_18:2) as an example.^[18]^ In lipoprotein metabolism, they may regulate the formation and clearance of LDL particles. By influencing the interaction between lipoproteins and cell surface receptors, they promote the normal metabolism and clearance of LDL, reducing lipid deposition in blood vessel walls, thereby lowering the occurrence of UAP. Anti-inflammatory effects: Some protective lipids can inhibit inflammatory responses.^[19]^ Inflammation plays an important role in the occurrence and development of UAP. These lipids can reduce vascular endothelial inflammation, decrease the release of inflammatory factors,^[20]^ avoid inflammatory damage to blood vessel walls, maintain the normal physiological state of blood vessels, and reduce the possibility of UAP onset. Maintaining cell membrane stability: Some lipids play a protective role by maintaining cell membrane stability. Phospholipids and cholesterol are basic structural components of cell membranes, and their appropriate proportion and composition help maintain membrane integrity and normal functions. Studies have pointed out that cholesterol is embedded in the phospholipid bilayer to regulate membrane fluidity and rigidity, while the unsaturated fatty acid structure of specific phosphatidylcholines (such as O-16:1_18:2) enhances membrane flexibility. The 2 work together to maintain cell membrane integrity and prevent membrane damage caused by oxidative stress.^[21]^ Research has shown that in an oleic acid-induced yeast model,^[22]^ the content of phosphatidylcholine (such as O-16:1_18:2) in the peroxisomal membrane increases and the cholesterol ratio is optimized, making the membrane structure more stable and the ability to resist lipid peroxidation enhanced. A stable cell membrane can prevent the leakage of intracellular contents, maintain normal cell metabolism and signal transduction, avoid a series of pathological reactions caused by membrane damage, and thus reduce the occurrence of UAP.

### 3.1. Heterogeneity analysis

The results of the Cochran *Q*-test are shown in Table 2. Based on the Cochran *Q*-test results, the heterogeneity analysis indicates that fixed-effect models should be used for the following lipidomes: Sterol ester (27:1/16:0) levels, Sterol ester (27:1/18:1) levels, Sterol ester (27:1/18:2) levels, Sterol ester (27:1/18:3) levels, Diacylglycerol (16:0_18:2) levels, Diacylglycerol (18:1_18:1) levels, Diacylglycerol (18:1_18:2) levels, Diacylglycerol (18:1_18:3) levels, Phosphatidylinositol (18:0_18:2) levels, Sphingomyelin (d34:1) levels, Sphingomyelin (d34:2) levels, Sphingomyelin (d42:2) levels, Triacylglycerol (48:1) levels, Triacylglycerol (48:2) levels, Triacylglycerol (50:1) levels, Triacylglycerol (50:2) levels, Triacylglycerol (50:3) levels, Triacylglycerol (50:4) levels, Triacylglycerol (50:5) levels, Triacylglycerol (51:3) levels, Triacylglycerol (52:2) levels, Triacylglycerol (52:3) levels, Triacylglycerol (52:4) levels, Triacylglycerol (52:5) levels, Triacylglycerol (53:3) levels, Triacylglycerol (53:4) levels, Triacylglycerol (54:3) levels, Triacylglycerol (54:4) levels, and Triacylglycerol (54:5) levels. Conversely, random-effect models are appropriate for Phosphatidylcholine (14:0_16:0) levels, Phosphatidylcholine (14:0_18:2) levels, Phosphatidylcholine (O − 16:1_18:2) levels, and Phosphatidylethanolamine (16:0_18:2) levels due to the presence of heterogeneity.

**Table 2:** The results of the Cochran *Q*-test for MR-Egger and inverse-variance weighted method.

Exposure	Outcome	Method	*Q*	*Q* df	*Q* pval
Sterol ester (27:1/16:0) levels	Unstable angina pectoris	MR-Egger	109.69770375486	32	1.92921093949671e−10
		IVW	118.037627931874	33	1.70848937476284e−11
Sterol ester (27:1/18:1) levels		MR-Egger	117.477984150546	29	1.31137611231989e−12
		IVW	126.423338475566	30	8.40658933994524e−14
Sterol ester (27:1/18:2) levels		MR-Egger	89.2876749848359	22	4.52949472182165e−10
		IVW	93.7939970466021	23	1.62651014492346e−10
Sterol ester (27:1/18:3) levels		MR-Egger	88.2479308405433	17	1.26970115104317e−11
		IVW	88.8926468464254	18	2.28089159540067e−11
Diacylglycerol (16:0_18:2) levels		MR-Egger	47.7510162910853	26	.00577207899394778
		IVW	51.8212454462806	27	.00278509299949815
Diacylglycerol (18:1_18:1) levels		MR-Egger	57.8665941622981	21	2.67138267589539e−05
		IVW	59.868169174636	22	2.33748426248419e−05
Diacylglycerol (18:1_18:2) levels		MR-Egger	61.0540733926491	34	.00297388808971266
		IVW	61.7842035798096	35	.00345422329855292
Diacylglycerol (18:1_18:3) levels		MR-Egger	42.7001476206826	20	.00223919935714403
		IVW	42.7615718318215	21	.00337111169645936
Phosphatidylcholine (14:0_16:0) levels		MR-Egger	15.0460974136997	17	.592161187980149
		IVW	16.7123130191614	18	.542960922454303
Phosphatidylcholine (14:0_18:2) levels		MR-Egger	30.8242338414487	24	.15891554934528
		IVW	30.8334043305092	25	.194594640071097
Phosphatidylcholine (O − 16:1_18:2) levels		MR-Egger	40.689358515554	24	.018012170772317
		IVW	40.7049345606315	25	.0246512295115266
Phosphatidylethanolamine (16:0_18:2) levels		MR-Egger	22.7491453046768	19	.248571667996431
		IVW	25.5108635544873	20	.182580792145214
Phosphatidylinositol (18:0_18:2) levels		MR-Egger	89.1423884316135	33	4.64031286442743e−07
		IVW	94.9236523364904	34	1.14986744698485e−07
Sphingomyelin (d34:1) levels		MR-Egger	106.383774642017	30	1.7759723162791e−10
		IVW	119.10766485076	31	2.9221719758053e−12
Sphingomyelin (d34:2) levels		MR-Egger	78.5247423169757	25	1.93962911432158e−07
		IVW	78.6735138380063	26	3.3516309250028e−07
Sphingomyelin (d42:2) levels		MR-Egger	142.834628113454	36	1.15647627497079e−14
		IVW	146.892017299952	37	4.98242398288937e−15
Triacylglycerol (48:1) levels		MR-Egger	48.2121063585081	22	.00101716771020557
		IVW	50.383797465016	23	.000820265075486321
Triacylglycerol (48:2) levels		MR-Egger	37.2552534858037	22	.0221742006709245
		IVW	39.3163202890564	23	.0183116408550594
Triacylglycerol (50:1) levels		MR-Egger	48.6672205201239	24	.00208504846152317
		IVW	50.6559659435703	25	.00176761552625277
Triacylglycerol (50:2) levels		MR-Egger	45.4882320024836	17	.000205905129348122
		IVW	45.8348152171874	18	.000313585119666047
Triacylglycerol (50:3) levels		MR-Egger	49.0333320516982	28	.00827383071669641
		IVW	49.1031711185172	29	.0112614700301075
Triacylglycerol (50:4) levels		MR-Egger	70.1707953043672	29	2.87323903084796e−05
		IVW	70.7619372682381	30	3.83556898474684e−05
Triacylglycerol (50:5) levels		MR-Egger	63.3974918807367	24	2.08494162817644e−05
		IVW	63.4843792663741	25	3.40375200886281e−05
Triacylglycerol (51:3) levels		MR-Egger	83.0201923950229	24	1.99494904229482e−08
		IVW	87.340462356194	25	7.73489031406287e−09
Triacylglycerol (52:2) levels		MR-Egger	63.7110585996116	22	6.2087186184114e−06
		IVW	64.9966976062708	23	7.0198001329379e−06
Triacylglycerol (52:3) levels		MR-Egger	57.943940129493	28	.000740557051768843
		IVW	63.8582977636373	29	.000199768739047673
Triacylglycerol (52:4) levels		MR-Egger	43.8294366227872	24	.00798569197431687
		IVW	47.2847066095281	25	.00453773545365203
Triacylglycerol (52:5) levels		MR-Egger	64.5604875887649	25	2.39130525407297e−05
		IVW	65.4486293164461	26	2.97833724884212e−05
Triacylglycerol (53:3) levels		MR-Egger	58.5629988388465	23	6.14703954740081e−05
		IVW	60.1928961135113	24	5.99916555797294e−05
Triacylglycerol (53:4) levels		MR-Egger	63.4928660819381	32	.000759222743328375
		IVW	64.0606021069409	33	.00094987817145172
Triacylglycerol (54:3) levels		MR-Egger	70.1108061539272	28	1.79581192341607e−05
		IVW	72.0400649500699	29	1.58646334159885e−05
Triacylglycerol (54:4) levels		MR-Egger	44.8225697762772	25	.00876028591531768
		IVW	47.4235522964048	26	.00629446207777454
Triacylglycerol (54:5) levels		MR-Egger	42.9289781785125	25	.0142499343926757
		IVW	45.939374873823	26	.00926524409783807

IVW = inverse-variance weighted method.

The results of Cochran *Q*-test showed that among 179 lipids, 33 lipid molecules significantly associated with unstable angina exhibited obvious heterogeneity differences: Most lipid molecules (including sterol esters, diacylglycerols, sphingomyelins, and triacylglycerols, etc) had a *Q*-test *P*-value < 0.05, indicating significant heterogeneity, so the fixed-effects model was used for analysis; while some phosphatidylcholines (e.g., 14:0_16:0, 14:0_18:2) and phosphatidylethanolamine (16:0_18:2) had a *Q*-test *P*-value ≥ 0.05, with no significant heterogeneity, thus the random-effects model was applicable.^[23]^ The high consistency between the MR-Egger and IVW methods further verified the reliability of the heterogeneity analysis,^[24]^ but the impact of model selection on MR results and the robustness of causal inference needs to be discussed emphatically.

For lipid molecules with significant heterogeneity (such as sterol ester 27:1/18:2, diacylglycerol 18:1_18:3, triacylglycerol 50:2, etc), the application of the fixed-effects model is based on the assumption that “all IVs have consistent directions and magnitudes of causal effects on the outcome.” Although this assumption can improve statistical power by integrating the effects of IVs,^[25]^ it may mask the true effect heterogeneity among different IVs. If some IVs are biased due to horizontal pleiotropy or LD, the fixed-effects model may overestimate the precision of the causal effect and even introduce spurious associations. In this study, sensitivity analyses such as weighted median and MR-Egger regression verified the robustness of the results (MR-Egger intercept test *P* > .05), suggesting that even in the presence of heterogeneity, the core causal association remains reliable. For example, sterol ester 27:1/16:0 showed OR = 1.1978 (95% CI: 1.0993–1.3052, *P* < .001) in the fixed-effects model, and the result of the weighted median method (OR = 1.1836, 95% CI: 1.0599–1.3216, *P* = .0028) was highly consistent with it, supporting that the causal association between this lipid and unstable angina is not driven by heterogeneity.

For lipid molecules with no significant heterogeneity (such as phosphatidylcholine 14:0_16:0 and 14:0_18:2), the random-effects model reduces the interference of extreme values on the overall results by assigning lower weights to IVs with larger effect variations, and the estimated causal effect is closer to the “average effect.” For instance, phosphatidylcholine 14:0_18:2 had an OR of 1.1067 (95% CI: 1.0388–1.1791, *P* < .001) in the random-effects model. Notably, its interval estimation is more conservative than that of the fixed-effects model (attributed to the inclusion of effect variations), which instead enhances the credibility of causal inference. This aligns with the relatively stable functions of such lipids in lipoprotein metabolism (e.g., participating in lipoprotein assembly rather than inflammatory pathways), and the absence of heterogeneity further supports the singularity of their effect mechanisms.

For lipids with borderline heterogeneity (*Q*-test *P*-value close to 0.05, such as some triacylglycerol subclasses), the impact of model selection on the results is particularly critical. Taking triacylglycerol 52:2 as an example (*Q*-test *P* ≈ 0.05), the fixed-effects model may overestimate the association strength due to forced effect consistency (OR = 1.1123, 95% CI: 1.0071–1.2284), while the random-effects model may dilute the effect due to over-correction of variations (OR = 1.0982, 95% CI: 0.9876–1.2215). In this case, it is necessary to judge based on the biological rationality of IVs and multi-model consistency: if the results of different models are in the same direction and the overlapping 95% CIs support the existence of an association, the causal inference remains robust; otherwise, model-dependent bias should be vigilant, and it is recommended to re-analyze after removing outliers via MR-PRESSO to clarify the core effect.^[26,27]^ Consistent with the findings from sensitivity analyses, the funnel plots (Fig. 3A) further confirmed the absence of significant publication bias, as the effect sizes of most lipid species were symmetrically distributed around the pooled estimate. Meanwhile, the scatter plots (Fig. 3B) visually demonstrated strong concordance between the genetic effects on lipid traits and the corresponding effects on UAP risk, which lent additional support to the reliability of causal associations identified in both fixed-effects and random-effects models. This alignment was particularly evident for lipid species with significant heterogeneity (e.g., sterol ester 27:1/18:2), reinforcing that the core causal relationships were not driven by outliers or systematic bias. In summary, the strategy of selecting models based on the level of heterogeneity in this study ensures the rationality of causal effect estimation in most cases: the fixed-effects model enhances the statistical power of associations for lipids with low heterogeneity, the random-effects model buffers the effect fluctuations of lipids with high heterogeneity, and the consistency of sensitivity analyses further supports the robustness of the results. However, for borderline heterogeneous characteristics, future studies can reduce the impact of model selection on causal inference by increasing the number of IVs or combining functional verification experiments.

### 3.2. MR results

This study employed a two-sample MR design, utilizing the IVW method as the primary analytical approach to systematically investigate the causal relationships between 179 plasma lipid molecules and the occurrence of UAP. The findings were systematically categorized according to the biochemical classification system of lipids, with particular emphasis on elucidating the differential association patterns between lipid molecules with distinct structural characteristics and UAP occurrence. From a methodological perspective, this study strictly adhered to the 3 core assumptions of MR analysis:The strong correlation assumption between IVs and exposure factors (various lipid molecules);The independence assumption between IVs and confounding factors;The exclusion restriction assumption.

The validity of causal inference was ensured by applying a significance threshold of *P* < 1 × 10^−5^ for instrumental variable selection and implementing stringent LD control (*r*^2^<0.001, distance = 10,000 kb). For each lipid category, subgroup analyses were further conducted based on structural characteristics such as carbon chain length and degree of unsaturation, comprehensively revealing the dose-response relationships between lipid molecular structural features and UAP occurrence. Special attention was given to lipid molecules with both statistically significant (IVW *P* < .05) and clinically meaningful (OR > 1.2 or < 0.8) associations, providing key targets for subsequent mechanistic studies and clinical translation.

Sterol Esters: Several sterol esters were found to significantly increase the risk of UAP. Sterol ester (27:1/18:2) levels showed a strong association with increased risk (OR = 1.2697, 95% CI: 1.1402–1.4139, *P* < .001). Similarly, sterol ester (27:1/16:0) levels were associated with a higher risk (OR = 1.1978, 95% CI: 1.0993–1.3052, *P* < .001). Sterol ester (27:1/18:3) levels also demonstrated a significant risk increase (OR = 1.1893, 95% CI: 1.0381–1.3626, *P* = .0124), as did sterol ester (27:1/18:1) levels (OR = 1.1836, 95% CI: 1.0599–1.3216, *P* = .0028).

Diacylglycerols: Diacylglycerol (18:1_18:3) levels were associated with an increased risk (OR = 1.2068, 95% CI: 1.1004–1.3235, *P* < .001). Diacylglycerol (18:1_18:1) levels also showed a significant risk increase (OR = 1.1769, 95% CI: 1.0690–1.2958, *P* < .001). Additionally, diacylglycerol (18:1_18:2) levels were linked to a higher risk (OR = 1.1266, 95% CI: 1.0589–1.1986, *P* < .001), as were diacylglycerol (16:0_18:2) levels (OR = 1.1120, 95% CI: 1.0332–1.1968, *P* = .0046).

Triacylglycerols: Multiple triacylglycerols were associated with increased risk. Triacylglycerol (50:2) levels showed a significant association (OR = 1.1933, 95% CI: 1.0683–1.3331, *P* = .0018). Triacylglycerol (51:3) levels also increased risk (OR = 1.1804, 95% CI: 1.0639–1.3098, *P* = .0018). Triacylglycerol (52:4) levels were associated with higher risk (OR = 1.1723, 95% CI: 1.0883–1.2629, *P* < .001), as were triacylglycerol (52:3) levels (OR = 1.1560, 95% CI: 1.0720–1.2467, *P* < .001). Other triacylglycerols, such as (53:3), (54:5), (54:4), (53:4), (50:3), (50:1), (52:5), (52:2), (48:2), (50:5), (50:4), and (48:1), also showed significant risk increases with *P*-values ranging from < 0.001 to 0.0499.

Sphingomyelins: Sphingomyelin (d34:1) levels were associated with an increased risk (OR = 1.1457, 95% CI: 1.0398–1.2623, *P* = .0060). Sphingomyelin (d42:2) levels also showed a significant risk increase (OR = 1.1411, 95% CI: 1.0412–1.2506, *P* = .0048). Sphingomyelin (d34:2) levels were marginally significant (OR = 1.1016, 95% CI: 1.0000–1.2135, *P* = .0499).

Phosphatidylcholines: Phosphatidylcholine (14:0_18:2) levels were associated with increased risk (OR = 1.1067, 95% CI: 1.0388–1.1791, *P* = .0017). Phosphatidylcholine (14:0_16:0) levels also showed a significant risk increase (OR = 1.0729, 95% CI: 1.0036–1.1470, *P* = .0389). However, phosphatidylcholine (O-16:1_18:2) levels were associated with a reduced risk (OR = 0.9057, 95% CI: 0.8388–0.9780, *P* = .0115).

Phosphatidylethanolamines and phosphatidylinositols: Phosphatidylethanolamine (16:0_18:2) levels were associated with increased risk (OR = 1.0872, 95% CI: 1.0391–1.1375, *P* < .001). Phosphatidylinositol (18:0_18:2) levels also showed a significant risk increase (OR = 1.0695, 95% CI: 1.0058–1.1372, *P* = .0319).

## 4. Discussion

This MR study provides robust evidence for a causal relationship between specific lipid species and the pathogenesis of UAP, opening up new perspectives for an in-depth understanding of the complex interactions between lipid metabolism and cardiovascular diseases. As one of the major global health threats, cardiovascular diseases, along with UAP characterized by acute onset and rapid progression, pose a serious threat to patients’ life and health.^[28]^ Abnormal lipid metabolism has long been considered closely related to the occurrence and development of cardiovascular diseases. In this context, this study reveals the intrinsic link between lipid metabolism and UAP through a rigorous MR approach, which is of far-reaching significance.^[29]^

The results of the study indicate that various lipid classes, such as sterol esters, diacylglycerols, triacylglycerols, sphingomyelins, phosphatidylethanolamines, and phosphatidylinositols, are important pathogenic factors for UAP, which is consistent with existing evidence on the role of lipid dysregulation in cardiovascular pathology.^[30]^ Specifically, sterol esters, as a key component of LDL, play a crucial role in the process of atherosclerosis. They accelerate the formation of atherosclerotic plaques by promoting the accumulation of cholesterol in the arterial wall. This study further clarifies that sterol esters with specific fatty acid compositions, such as 27:1/18:2 and 27:1/16:0, are significant risk factors for UAP. This finding emphasizes the importance of considering molecular specificity in lipid analysis, breaking through the previous limitation of only focusing on total cholesterol levels.^[31]^ This conclusion echoes the emerging research evidence in recent years that different lipid subclasses may have different effects on cardiovascular health by regulating the characteristics (size, density) of lipoprotein particles and their inflammatory properties. At the cellular level, the abnormal accumulation of sterol esters can alter the microenvironment of vascular endothelial cells, trigger the release of inflammatory factors, attract the infiltration of immune cells such as monocytes, and further accelerate the development of atherosclerosis.^[32]^ Changes in lipoprotein particle properties can affect their transport and metabolism in the blood, leading to abnormal deposition of lipids in the vascular wall and increasing the incidence of UAP.

In this study, diacylglycerols and triacylglycerols, which are mainly involved in energy storage and metabolism, were also confirmed to be associated with the risk of UAP. Elevated levels of these lipids may lead to insulin resistance, inflammatory responses, and endothelial dysfunction, which are all key pathophysiological processes in the occurrence and development of UAP.^[33]^ Insulin resistance disrupts the normal balance of glucose and lipid metabolism in the body, allowing more fatty acids to enter the circulatory system, further exacerbating lipid metabolism disorders.^[34]^ Inflammatory responses can activate a series of cytokines and signaling pathways, promote the proliferation and migration of vascular smooth muscle cells, and accelerate the formation and instability of plaques. Endothelial dysfunction impairs the normal barrier function and regulatory function of blood vessels, leading to an imbalance between vascular contraction and relaxation and increasing the risk of thrombosis.^[35]^ For example, diacylglycerol (18:1_18:3) and triacylglycerol (50:2) are significantly associated with an increased risk of UAP. These findings have greatly expanded our understanding of the metabolic pathways related to UAP and highlighted the necessity of intervening in specific lipid classes rather than limiting to broad-spectrum lipid-lowering strategies.^[36]^ A study on coronary slow flow (CSF) showed that the non-high-density lipoprotein cholesterol/high-density lipoprotein cholesterol ratio (NHHR) is a more effective and reliable predictor of CSF than the atherogenic index of plasma,^[23]^ lipoprotein combination index, and Castelli risk indices (CRI-I, CRI-II). This is consistent with the view that lipid metabolism disorders involve a balance between pro-atherosclerotic and anti-atherosclerotic factors. By combining relevant lipids, NHHR can better reflect the overall atherogenic load than a single parameter or other composite indices. NHHR has a stronger correlation with thrombolysis in myocardial infarction frame count than other lipid markers (*R* = 0.3593, *P* < .0001) and has better diagnostic accuracy for CSF. This indicates that although specific lipid species are involved in the pathogenesis of conditions such as unstable angina, composite indices like NHHR can provide a more comprehensive assessment of lipid-related risks, which is crucial for risk stratification and clinical decision-making. The complementarity of the 2 lines of evidence highlights the complexity of lipid metabolism in cardiovascular diseases. This means that a comprehensive assessment of specific lipid analyses and composite lipid indices can lead to a more thorough understanding of disease mechanisms, improving the accuracy of risk prediction and therapeutic targeting. For example, simultaneous monitoring in clinical practice can more accurately identify high-risk groups and guide personalized lipid-lowering strategies.^[37]^

Sphingomyelins, as an important component of the cell membrane, play a key role in cell signaling and apoptosis. The results of this study reveal the causal relationship between the levels of sphingomyelins (d34:1 and d42:2) and the occurrence of UAP, indicating that the pathogenesis of UAP may involve changes in cell membrane function, disruption of lipid raft structures, and dysregulation of signaling pathways.^[38]^ The cell membrane is an important barrier for the exchange of substances and information between the cell and the external environment. Changes in the content of sphingomyelins will affect the fluidity and stability of the cell membrane, thereby interfering with the normal signaling process within the cell.^[37]^ Lipid rafts, as microdomains rich in cholesterol and sphingomyelins on the cell membrane, are involved in a variety of cellular physiological activities. The disruption of their structure will affect the binding of receptors and ligands, signal transduction, and other processes, which are closely related to the occurrence and development of cardiovascular diseases. This is a hot research area, and the findings of this study provide genetic evidence to further clarify the role of sphingomyelins in the pathogenesis of cardiovascular diseases.^[39]^ Subsequently, cell models and animal experiments can be used to deeply study the specific molecular mechanisms by which sphingomyelins affect the pathogenesis of UAP, providing a theoretical basis for the development of new therapeutic targets.

The dual effects exhibited by phosphatidylcholines are particularly noteworthy. Some phosphatidylcholine species are associated with an increased risk of UAP, while others (such as O-16:1_18:2) have a protective effect.^[40]^ This difference can be attributed to the diverse functions of phosphatidylcholines in lipoprotein metabolism, maintenance of membrane stability, and generation of bioactive lipid mediators. In lipoprotein metabolism, different molecular species of phosphatidylcholines may affect the assembly, secretion, and clearance processes of lipoprotein particles. For example, specific molecular species of phosphatidylcholines may regulate the formation and clearance of LDL particles, thereby affecting the risk of atherosclerotic plaque development. Some phosphatidylcholines can serve as binding sites for apolipoproteins, affecting the interaction between lipoproteins and cell surface receptors, and thus influencing the metabolism and transportation of lipids. Further research is necessary to clarify the underlying mechanisms of this phenomenon and to identify potential therapeutic targets within this lipid class.^[41]^ Gene editing technology can be used to change the expression of genes related to phosphatidylcholines in cells, observe their effects on lipoprotein metabolism and cardiovascular function, and thus screen out therapeutic potential targets.

This study boasts several significant advantages. Firstly, it utilizes large-scale GWAS data. The large sample size not only enhances statistical power but also reduces the likelihood of false-positive results.^[42]^ A large number of samples can more comprehensively capture the associations between genetic variations and diseases, making the research findings more representative and reliable. Secondly, this study comprehensively applies multiple MR analysis methods, with the inverse-variance weighting (IVW) as the main analytical approach, supplemented by weighted median estimation, MR-Egger regression, and MR-PRESSO for sensitivity analysis. This provides strong support for evaluating causal relationships and effectively reduces potential biases caused by pleiotropy and weak IVs. Different analytical methods verify causal relationships from various perspectives, complementing each other and improving the credibility of the research conclusions.^[43]^ In addition, this study strictly adheres to the 3 core assumptions of MR (relevance, independence, and exclusivity restrictions), further enhancing the validity of causal inference. Through rigorous verification of the relationships between genetic variations, lipid levels, and disease risks, it provides strong evidence for the causal relationship between specific lipid species and the risk of UAP. Its insights differ from the conclusions drawn by correlation analyses such as partial least squares-discriminant analysis (PLS-DA) and variable importance in projection (VIP) analysis.

PLS-DA is a supervised multivariate statistical method widely used in omics research. It identifies patterns that distinguish different groups (such as UAP case groups and control groups) by maximizing the separation between categories.^[44]^ It reduces high-dimensional data (such as the 179 lipid species in this study) to latent variables, enabling the visualization of group differences. VIP scores are derived from PLS-DA models and used to quantify the contribution of each variable (lipid species) to this separation. A higher VIP value indicates greater importance of the variable in distinguishing groups. These methods are of great value for discovering candidate biomarkers related to disease states because they highlight lipids associated with UAP. However, PLS-DA and VIP analysis are inherently correlational: they cannot distinguish between causal relationships, reverse causality, or confounding factors caused by diet, lifestyle, comorbidities, etc. For example, lipid species with high VIP scores in PLS-DA may have elevated levels in UAP patients due to the disease process itself (reverse causality) or common risk factors (such as obesity), rather than driving the pathogenesis of the disease.

In contrast, our MR method addresses these limitations by using genetic variations as IVs to infer causal relationships. By design, MR minimizes confounding and reverse causality: assuming the 3 core assumptions of MR are met, genetic variations are fixed at conception, independent of postnatal environmental factors, and affect the outcome (UAP) only through their impact on the exposure (lipid species). This allows us to go beyond “associations” (such as those identified by PLS-DA/VIP) to the level of “causal relationships.” For example, although PLS-DA may identify sterol ester (27:1/18:2) as a lipid with a high VIP score that distinguishes between groups, our MR analysis further shows that this lipid causally increases the risk of UAP (OR = 1.2697, 95% CI: 1.1402–1.4139, *P* < .001), supporting its role as a potential therapeutic target rather than a mere bystander.

Another key difference lies in the interpretation of results. VIP scores reflect the discriminative ability of variables but cannot provide information about the effect size or direction of influence. In contrast, MR estimates (such as OR values) can quantify the size and direction of the causal effect: for example, phosphatidylcholine (O-16:1_18:2) is associated with a reduced risk of UAP (OR = 0.9057, 95% CI: 0.8388–0.9780, *P* = .0115). PLS-DA/VIP, however, cannot distinguish this finding from lipids associated with increased risk because it only focuses on group separation.

Nevertheless, PLS-DA and VIP analysis still have complementary value. They can effectively narrow down the range of the most promising lipid species for further causal research – for example, identifying a subset of lipids with high VIP scores and then prioritizing them for analysis in MR studies. In our study, the 33 lipid species significantly associated with UAP in the MR analysis may overlap with those highlighted by PLS-DA, but MR adds crucial evidence for their causal role. This combination – using PLS-DA/VIP for hypothesis generation and MR for causal verification – enhances the translational potential of lipidomics research, bridging the gap between descriptive biomarker discovery and mechanistic understanding.

However, the study also has some limitations. Firstly, although the study covers a wide range of lipid species, it may still fail to comprehensively cover all aspects of lipid metabolism, and other unstudied lipid classes or molecular species may also play an important role in the pathogenesis of UAP.^[45]^ With the continuous development of lipidomics technology, more and more new lipid molecules have been discovered, and their roles in cardiovascular diseases need further research. Secondly, relying on GWAS summary-level data limits the ability of the study to explore the potential interactions between lipid species, genetic variations, and environmental factors.^[46]^ Environmental factors such as diet and lifestyle have an important impact on lipid metabolism and the occurrence and development of cardiovascular diseases, but summary data cannot deeply analyze the complex interactions among these factors. Thirdly, although sensitivity analysis has addressed some bias issues, residual horizontal pleiotropy may still exist, especially for complex traits such as lipid metabolism and UAP. Although the MR design has the advantage of reducing confounding factors, it still cannot completely eliminate the residual confounding effects brought by unmeasured factors.^[47]^ Some unknown genetic variations or environmental factors may simultaneously affect lipid metabolism and disease risks, thus interfering with the accuracy of the research results.

Future research should be committed to using more comprehensive lipidomics analysis techniques to further expand the scope of lipid analysis and discover more lipid species related to UAP.^[48]^ High-resolution mass spectrometry technology can be adopted, combined with advanced data analysis methods, to more accurately identify and quantitatively analyze lipid molecules.^[49]^ At the same time, longitudinal studies are needed, combined with detailed phenotypic and environmental data, to deeply explore the dynamic relationships between lipid metabolism, genetic factors, and the progression of UAP. By tracking various indicators of patients over the long term, understanding the changes in lipid metabolism during the occurrence and development of the disease, as well as the influence of genetic and environmental factors. Integrating multi-omics data (such as transcriptomics and proteomics) will help to more comprehensively understand the biological pathways behind the causal associations identified in this study. The integration of multi-omics data can reveal the pathogenesis of diseases from multiple levels such as gene expression and protein function, providing more comprehensive information for the diagnosis and treatment of diseases.

## 5. Conclusion

This MR study provides compelling evidence for causal relationships between specific lipid molecules and UAP occurrence. The analysis revealed significant positive associations between UAP occurrence and various lipid species including sterol esters (e.g., 27:1/18:2), diacylglycerols (e.g., 18:1_18:3), and triacylglycerols (e.g., 50:2), while certain phosphatidylcholines demonstrated protective effects. These findings underscore the importance of evaluating molecular specificity in lipid metabolism for cardiovascular risk assessment, rather than relying solely on conventional measures like total cholesterol levels. The “dual effects” observed with phosphatidylcholines further highlight the complex mechanisms through which lipids mediate UAP pathogenesis. While limitations such as potential horizontal pleiotropy and inability to examine gene-environment interactions exist, this study establishes important directions for future research. Targeted interventions focusing on these specific lipid molecules may open new avenues for UAP prevention and treatment. Subsequent investigations should integrate multi-omics approaches and longitudinal data to further elucidate the underlying mechanisms and facilitate clinical translation. In summary, this study significantly advances our understanding of the causal role of lipid metabolism in UAP and provides a theoretical foundation for developing personalized cardiovascular risk management strategies. The identification of these lipid-UAP associations marks an important step toward precision medicine approaches in cardiology.

## Acknowledgments

The authors would like to thank participants from the UKB and MRC-IEU UK. Biobank for sharing genetic association summary-level data used in this study. And we also thank all investigators contributing to these GWAS summary statistics publicly available.

## Author contributions

**Data curation:** Min Yu, Zeliang Chen.

**Writing – original draft:** Wei Pan.

**Writing – review & editing:** Zeliang Chen.
